# Predictive Model of Lymphocyte-Specific Protein Tyrosine Kinase (LCK) Autoregulation

**DOI:** 10.1007/s12195-016-0438-7

**Published:** 2016-04-26

**Authors:** Jennifer A. Rohrs, Pin Wang, Stacey D. Finley

**Affiliations:** 1 Department of Biomedical Engineering, University of Southern California, 1042 Downey Way, DRB 140, Los Angeles, CA 90089 USA; 2 Mork Family Department of Chemical Engineering and Materials Science, University of Southern California, Los Angeles, CA USA

**Keywords:** Systems biology, Computational modeling, T cell signaling, Parameter estimation

## Abstract

Lymphocyte-specific protein tyrosine kinase (LCK) is a key activator of T cells; however, little is known about the specific autoregulatory mechanisms that control its activity. We have constructed a model of LCK autophosphorylation and phosphorylation by the regulating kinase CSK. The model was fit to existing experimental data in the literature that presents an *in vitro* reconstituted membrane system, which provides more physiologically relevant kinetic measurements than traditional solution-based systems. The model is able to predict a robust mechanism of LCK autoregulation. It provides insights into the molecular causes of key site-specific phosphorylation differences between distinct experimental conditions. Probing the model also provides new hypotheses regarding the influence of individual binding and catalytic rates, which can be tested experimentally. This minimal model is required to elucidate the mechanistic interactions of LCK and CSK and can be further expanded to better understand T cell activation from a systems perspective. Our computational model enables the evaluation of LCK protein interactions that mediate T cell activation on a more quantitative level, providing new insights and testable hypotheses.

**Electronic supplementary material:**

The online version of this article (doi:10.1007/s12195-016-0438-7) contains supplementary material, which is available to authorized users.

## Introduction

Lymphocyte-specific protein tyrosine kinase (LCK) is a key regulator of T cell activation and differentiation.^6^,^38^ LCK helps to activate healthy T cells against diseased cells in the body by phosphorylating immunotyrosine activating motifs (ITAMS) on the CD3ζ chain of the T cell receptor (TCR).^25^ Mutations in the LCK gene can lead to autoimmune disease^14^ and contribute to cancer.^7^ Recently, LCK has been shown to play an important and complex role in the activation of chimeric antigen receptor (CAR) engineered T cells.^22^ CARs are engineered proteins that contain a variety of T cell signaling domains linked to an extracellular antibody single chain variable fragment (scFv). These proteins can activate T cells against a tumor-associated antigen to eradicate cancer cells.^22^,^37^ As CARs are adapted and modified to more specifically target different types of cancer cells, understanding the detailed mechanisms that govern their activation has become more important. Despite its strong role in regulating T cell signaling, little is known about the specific mechanisms that control LCK catalytic activity.

LCK is a multi-domain protein that can catalyze the phosphorylation of many substrates in T cells, including itself. LCK has two main phosphorylation sites, the tyrosine residues Y394 and Y505. Y394 is located close to the kinase domain, and, therefore, has been shown to play a significant role in substrate specificity.^23^ Y505 is located near the C-terminal tail of the protein. When phosphorylated, this tail is thought to fold up and bind in *cis*, locking the molecule in a “closed” conformation.^9^ Therefore, it is commonly accepted that phosphorylation at Y394 (denoted as LCK species P_394_U_505_) increases the catalytic activity of LCK and phosphorylation at Y505 (species U_394_P_505_) decreases catalytic activity.^44^ It has been shown that the unphosphorylated and doubly phosphorylated forms of LCK (species U_394_U_505_ and P_394_P_505_, respectively) retain an intermediate catalytic activity when acting on some substrates,^15^ although they may have more complex kinetics on others. These four forms of LCK distribute and aggregate differently within cells,^34^ and, while all four forms exist in resting T cells, efforts to calculate the exact ratios of the species have been inconclusive.^2^,^30^

Several proteins have been shown to control LCK phosphorylation. For example, C-terminal Src kinase (CSK) is a regulatory kinase that phosphorylates LCK specifically at Y505.^41^ In addition, several phosphatases act on LCK and CSK, most notably CD45 and PTPN22.^45^ It is commonly accepted that LCK can autophosphorylate at Y394,^44^ but it has only recently been appreciated that LCK can also autophosphorylate at Y505.^15^

The kinetics of these LCK phosphorylation and dephosphorylation reactions determine the pool of catalytically active LCK available to control T cell activation *in vivo*. Traditionally, the kinetics of these reactions are studied experimentally with recombinant proteins in solution^3^,^33^; however, inside the cell, LCK is largely bound to the plasma membrane, in a two-dimensional density distribution.^18^,^46^ This binding to the plasma membrane can profoundly influence a protein’s kinetics in several ways: (i) by altering the conformation of the protein, opening or closing available binding pockets, (ii) by changing the diffusion kinetics, which can alter the rate at which the enzyme encounters its substrate, and (iii) by altering the spatial segregation of certain groups of proteins in densely packed membranes, which may alter the ratio of active to inactive molecules in the system.^4^ Indeed, Hui *et al.* showed that the kinetics of LCK phosphorylation are vastly different when LCK is able to autophosphorylate on a membrane surface compared to in solution. These differences are both qualitative, in the order of phosphorylation of the two sites, and quantitative, in the rates of phosphorylation.^15^

In order to better understand the mechanisms through which LCK is regulated on the cell membrane, we have developed a computational model of LCK autophosphorylation and phosphorylation by the regulating kinase CSK. The model is fit to experimental data from the two-dimensional reconstituted membrane system developed by Hui and Vale.^15^ This data uses two concentrations of LCK: 500 molecules/μm^2^, which corresponds to a physiological level of LCK in the cell, and 50 molecules/μm^2^. One concentration of CSK is used, 500 molecules/μm^2^, which is slightly higher than the maximal amount of CSK present in the cells. Modeling this minimal system will allow us to predict the fundamental mechanisms of LCK activation and improve our understanding of the differences between two- and three-dimensional enzyme kinetics. Several computational models have been developed to study the early phosphorylation events in T cell activation^1^,^29^,^40^; however, none of them have accounted for the different species of LCK or the effects that the various catalytic and binding activities of these different species will have on T cell activation. The model of LCK activation that we have developed provides a basis for understanding LCK phosphorylation and catalytic activity and can be implemented in larger models of T cell signaling. Thus our work will enable a better understanding of how LCK autoregulation affects the control of T cell activation in the context of TCR antigen discrimination and CAR signaling.

## Materials and Methods

### Data Extraction

Experimental data was extracted from Hui and Vale^15^ using the MATLAB GRABIT program (The MathWorks Inc., Natick, MA). To correspond to the data, all simulations were normalized by the simulated amount of LCK at 90 min.

### Model Structure

Several different models were tested to find the simplest mechanism that is able to reproduce the data. Each model was progressively more complex. First, a mechanism in which the LCK phosphorylation events are directly catalyzed without a binding step to form intermediate dimers, a model described by 18 kinetic parameters, was implemented. Subsequently, a Michaelis–Menten mechanism in which the individual LCK enzymatic species have the same catalytic rate regardless of the substrate, a model involving 25 kinetic parameters, was implemented. Neither of these models was able to both reproduce training data used for parameter estimation and predict data not used in the fitting process (results not shown).

The third model implemented a Michaelis–Menten mechanism in which all of the LCK species have different binding and catalytic rates. This mechanism was able to fit the training data and predict new data. In this model, it is assumed that the phosphorylation events are primarily governed by two main factors: the strength of the interaction between the enzyme-substrate pair (i.e., the dissociation constant, *k*_*d*_), and the catalytic rate of the enzyme on the substrate. The dissociation constant is the dissociation rate (*K*_*off*_) divided by the association rate (*K*_*on*_). To avoid over parameterizing the model, we assume the association rate to be the same for all of the 16 LCK pairs, reducing the number of LCK binding parameters from 32 to 17. In Michaelis–Menten kinetics, the catalytic rate is generally the rate limiting step, so *K*_*on*_ was kept constant for all LCK–LCK or LCK–CSK binding pairs, as it is not expected to be rate limiting. The simplification of the association rates is also supported by studies showing that *K*_*on*_ generally falls within a relatively small range (about one order of magnitude) for many different protein interactions.^31^,^39^ However, we still allow the *k*_*d*_ values to differ between the LCK dimers by implementing a different *K*_*off*_ for each pair. By estimating the same association rate and different dissociation rates for the different LCK dimers, each binding pair can remain bound for different amounts of time depending on the strength of the individual interactions, allowing the *k*_*d*_ to span its full physiologically relevant range (more than 10 orders of magnitude).^26^,^32^

### Numerical Implementation of the Model

The model used to fit the training data is comprised of 23 non-linear ordinary differential equations (ODEs) (Supplementary File 1), and the model used for generating predictions with the catalytically inactive LCK is composed of 73 ODEs. The equations were written as a set of rules in BioNetGen,^11^ and implemented in MATLAB (The MathWorks Inc., Natick, MA). The model BioNetGen file is provided in Supplementary File 2, and the catalytically inactive model BioNetGen file is provided in Supplementary File 3.

### Parameter Estimation

Binding, on and off, and catalytic rates were estimated in an unbiased approach to find parameter sets that could both qualitatively differentiate between the rate of phosphorylation of Y394 and Y505 of LCK and quantitatively provide the best fit to the data.

Due to the large number of parameters to be fit, 38, and a lack of prior information about their possible ranges, a two-step approach was used to fit the model. First, a series of parameter sets was calculated by minimizing the weighted sum of the squared residual for a hybrid objective function (WSSR_hybr_) that accounts for both the quantitative fit to the data and the qualitative order of the phosphorylation curves of the two substrate sites (Y394 and Y505).^20^ Without the addition of the qualitative order of the curves in the hybrid WSSR, all of the optimal parameter sets over-fit the conditions in which Y394 is phosphorylated faster than Y505 (High LCK, High LCK + CSK, and Low LCK) without capturing the increase in Y505 phosphorylation in the Low LCK + CSK case. Parameter sets that did not capture that increase were penalized by having a higher WSSR_hybr_.

The WSSR_hybr_ is calculated by adding the WSSR for each data point to the WSSR for the distance between the Y394 and Y505 curves in each experimental condition:

$$\hbox{min} \left( {WSSR_{hybr} \left( \theta \right)} \right) = \hbox{min} \left( {\mathop \sum \limits_{i = 1}^{n} \left[ {W_{i}^{Y394} \left( {C_{exp,i}^{Y394} - C_{sim,i}^{Y394} \left( \theta \right)} \right)} \right]^{2} + \mathop \sum \limits_{i = 1}^{n} \left[ {W_{i}^{Y505} \left( {C_{exp,i}^{Y505} - C_{sim,i}^{Y505} \left( \theta \right)} \right)} \right]^{2} + \mathop \sum \limits_{i = 1}^{n} \left[ {W_{i}^{diff} \left( {\left( {C_{exp,i}^{Y394} - C_{exp,i}^{Y505} } \right) - \left( {C_{sim,i}^{Y394} \left( \theta \right) - C_{sim,i}^{Y505} \left( \theta \right)} \right)} \right)} \right]^{2} } \right)$$

where $C_{exp,i}^{Y394}$ and $C_{exp,i}^{Y505}$ are the *i*th experimentally measured LCK phosphorylation data point for Y394 or Y505, respectively. $C_{sim,i}^{Y394}$ and $C_{sim,i}^{Y505}$ are the simulated LCK phosphorylation at the *i*th time point. $W_{i}^{Y394}$, $W_{i}^{Y505}$, and $W_{i}^{diff}$ are weighting terms, taken as 1/$C_{exp,i}^{Y394}$, 1/$C_{exp,i}^{Y505}$, and $1/\left( {C_{\exp ,i}^{Y394} - C_{\exp ,i}^{Y505} } \right)$, respectively. *n* is the total number of experimental measurements. The minimization is subject to the upper and lower bounds of the free parameters, *θ*.

Particle swarm optimization (PSO)^17^ was used to find 1000 parameter sets that reached a minimum in the WSSR for the hybrid objective function. Briefly, PSO is able to efficiently search a parameter space by mimicking the ways in which groups of animals make decisions, for example how a colony of bees finds a new nesting site. Many particles move around the parameter space communicating their WSSR at each position. With each iteration, the positions and velocities of the particles are updated such that they approach a minimal WSSR. We used 31 particles searching a 38-dimensional bounded parameter space, with the particles starting at random points in the parameter space. Each iteration, a WSSR is calculated for every particle and the particles’ positions and velocities are then updated based on the their current WSSR and the global minimal WSSR. The algorithm is terminated when the global minimum WSSR remains constant for 50 iterations.

Next, the parameter sets from the hybrid WSSR were tailored to fit a quantitative WSSR (WSSR_quant_). The 1000 hybrid parameter sets were then used in the second step as inputs to a more local parameter estimation approach, performed by the MATLAB lsqnonlin function. This algorithm solves the non-linear least squares problem using the trust-region-reflective optimization algorithm, minimizing the WSSR_quant_:

$$\hbox{min} \left( {WSSR_{quant} \left( \theta \right)} \right) = \hbox{min} \left( {\mathop \sum \limits_{i = 1}^{n} \left[ {W_{i}^{Y394} \left( {C_{exp,i}^{Y394} - C_{sim,i}^{Y394} \left( \theta \right)} \right)} \right]^{2} + \mathop \sum \limits_{i = 1}^{n} \left[ {W_{i}^{Y505} \left( {C_{exp,i}^{Y505} - C_{sim,i}^{Y505} \left( \theta \right)} \right)} \right]^{2} } \right)$$

where the variables are the same as those used in the WSSR_hybr_ function.

### Clustering

Parameter set clustering was done using the MATLAB kmeans function. The optimal number of clusters was determined using the silhouette method.^36^ The silhouette plots measure the confidence that a given point lies in the cluster to which it is assigned, with each point getting a score from −1 to 1. We used the sum of the silhouette plot to calculate the optimal number of clusters, which was found to be three.

### Sensitivity Analysis

The extended Fourier amplitude sensitivity test (eFAST), a global variance-based sensitivity analysis, was used to understand how different parameters (“model inputs”) affect model predictions (“model outputs”). This method has been used previously to analyze computational biological models.^13^,^24^ In this method, the values of all of the inputs are varied together at different frequencies within a specified range and the model outputs are recorded. We varied the parameters 100-fold up and down from their median values, shown in Table 1. The Fourier transform of the output indicates which parameter frequencies contribute most, thus, which parameters are most sensitive. Varying all of the parameters together allows us to calculate two different indices of sensitivity: the first-order FAST indices, *Si*, a measurement of the local sensitivity of individual inputs, and the total FAST indices, *STi*, a measurement of the global sensitivity which accounts for second and higher-order interactions between multiple inputs. A greater total index than first-order index indicates that an input is more important in combination with other parameters than alone. We implemented the eFAST method using MATLAB code developed by Kirschner and colleagues.^27^

**Table 1:** Reacting species and parameters.

Enzyme	Substrate*	Dissociation rate (s^−1^): Median, (90% CI^#^)		Catalytic rate (s^−1^): Median, (90% CI)	
U_394_U_505_	**U**_**394**_U_505_	k_off,1_	1.0 × 10^−1^, (4.8 × 10^−7^–3.0 × 10^−2^)	k_cat,1_	2.7 × 10^3^, (2.1 × 10^3^–6.5 × 10^3^)
U_394_U_505_	U_394_ **U**_**505**_	k_off,2_	4.9 × 10^2^, (4.1 × 10^2^–1.5 × 10^3^)	k_cat,2_	4.2 × 10^1^, (2.9 × 10^1^–8.4 × 10^1^)
U_394_U_505_	P_394_ **U**_**505**_	k_off,3_	6.1 × 10^5^, (9.7 × 10^4^–2.8 × 10^6^)	k_cat,3_	7.6 × 10^−11^, (2.5 × 10^−11^–5.2 × 10^−8^)
P_394_U_505_	**U**_**394**_U_505_	k_off,4_	2.6 × 10^7^, (4.6 × 10^3^–2.8 × 10^7^)	k_cat,4_	2.2 × 10^−3^, (5.1 × 10^−6^–1.3 × 10^−1^)
P_394_U_505_	U_394_ **U**_**505**_	k_off,5_	6.4 × 10^2^, (5.5 × 10^2^–5.1 × 10^3^)	k_cat,5_	3.9 × 10^1^, (5.4 × 10^−1^–5.0 × 10^1^)
U_394_U_505_	**U**_**394**_P_505_	k_off,6_	7.6 × 10^−4^, (2.6 × 10^−5^–1.5 × 10^−3^)	k_cat,6_	4.6 × 10^−6^, (1.2 × 10^−8^–6.6 × 10^−6^)
U_394_P_505_	**U**_**394**_U_505_	k_off,7_	1.1 × 10^−3^, (1.2 × 10^−3^–1.8 × 10^−2^)	k_cat,7_	5.9 × 10^−12^, (8.3 × 10^−12^–3.8 × 10^−10^)
U_394_P_505_	U_394_ **U**_**505**_	k_off,8_	1.2 × 10^−3^, (1.9 × 10^−3^–6.1 × 10^−2^)	k_cat,8_	6.6 × 10^−11^, (1.0 × 10^−11^–1.8 × 10^−9^)
P_394_U_505_	P_394_ **U**_**505**_	k_off,9_	2.3 × 10^2^, (1.8 × 10^2^–7.0 × 10^2^)	k_cat,9_	1.3 × 10^1^, (9.4 × 10^0^–3.0 × 10^1^)
U_394_P_505_	P_394_ **U**_**505**_	k_off,10_	5.3 × 10^1^, (1.3 × 10^0^–7.7 × 10^3^)	k_cat,10_	6.3 × 10^−8^, (1.9 × 10^−8^–2.2 × 10^−3^)
P_394_U_505_	**U**_**394**_P_505_	k_off,11_	7.7 × 10^4^, (1.3 × 10^0^–2.1 × 10^4^)	k_cat,11_	9.5 × 10^−3^, (2.3 × 10^−5^–5.4 × 10^−1^)
U_394_P_505_	**U**_**394**_P_505_	k_off,12_	5.4 × 10^1^, (1.0 × 10^1^–1.1 × 10^4^)	k_cat,12_	1.9 × 10^−6^, (3.4 × 10^−7^–1.0 × 10^−4^)
P_394_P_505_	**U**_**394**_U_505_	k_off,13_	1.6 × 10^1^, (1.8 × 10^−3^–5.1 × 10^1^)	k_cat,13_	9.2 × 10^2^, (2.0 × 10^2^–9.1 × 10^3^)
P_394_P_505_	U_394_ **U**_**505**_	k_off,14_	5.6 × 10^6^, (1.1 × 10^6^–9.1 × 10^6^)	k_cat,14_	5.8 × 10^−11^, (3.0 × 10^−12^–6.5 × 10^−9^)
P_394_P_505_	P_394_ **U**_**505**_	k_off,15_	2.4 × 10^−11^, (1.8 × 10^−11^–9.7 × 10^−10^)	k_cat,15_	8.1 × 10^−4^, (8.1 × 10^−4^–8.1 × 10^−4^)
P_394_P_505_	**U**_**394**_P_505_	k_off,16_	1.6 × 10^0^, (6.8 × 10^−1^–2.5 × 10^0^)	k_cat,16_	6.3 × 10^−2^, (9.0 × 10^−5^–6.6 × 10^−2^)
CSK	U_394_ **U**_**505**_	k_off,CSK-UU_	4.4 × 10^−2^, (2.5 × 10^−2^–8.6 × 10^−2^)	k_cat,CSK-UU_	2.1 × 10^−3^, (1.5 × 10^−3^–2.0 × 10^−3^)
CSK	P_394_ **U**_**505**_	k_off,CSK-PU_	1.3 × 10^−6^, (1.4 × 10^−7^–1.4 × 10^−5^)	k_cat,CSK-PU_	1.8 × 10^7^, (9.3 × 10^5^–2.6 × 10^7^)

Parameter values represented as the median of 20 best-fit parameter sets

* Substrate site shown in bold

^#^ Confidence Interval

### Statistical Analysis

All statistical analyses were determined with a one-way analysis of variance (ANOVA) using Graphpad Prism version 6 for Mac (GraphPad Software, San Diego, CA).

## Results

### Model Construction

We have constructed a model of LCK autophosphorylation and phosphorylation by the kinase CSK. Below, we describe the salient features of the model, and full details are provided in the Methods section. Hui *et al.* experimentally proved that LCK, starting from a pool of unphosphorylated LCK, is able to phosphorylate itself in *trans*, and that this is the predominant form of phosphorylation.^15^ Accordingly, our model assumes that each LCK substrate site, Y394 and Y505, must be phosphorylated by a catalytic site on a different LCK molecule. This is done by implementing a Michaelis–Menten mechanism in which each pair of enzyme and substrate LCK species has different binding and catalytic rates. This model is characterized by 38 kinetic parameters.

**Figure 1:**
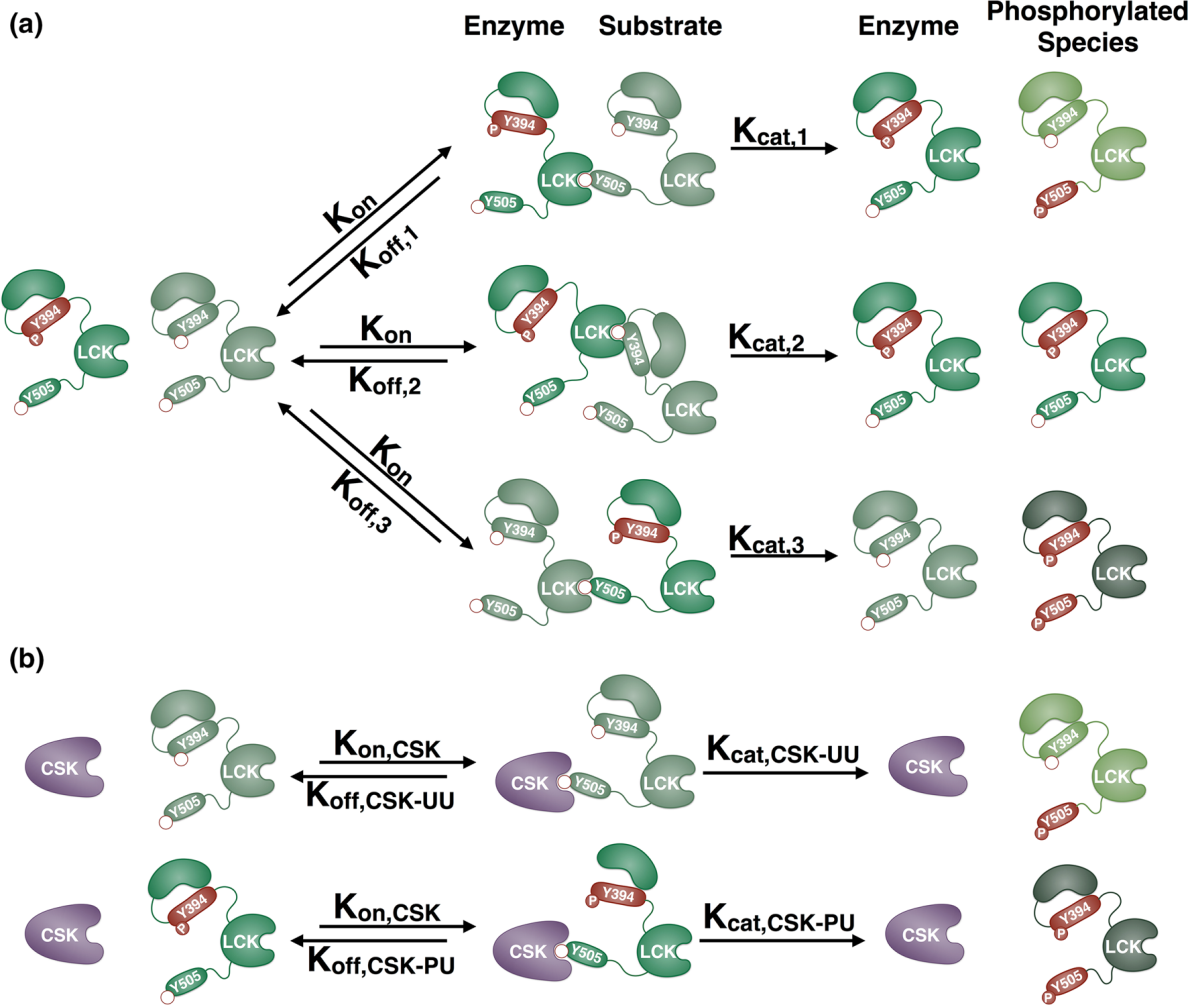
Schematic of LCK interactions. (a) The possible interactions between a representative pair of LCK species, U_394_U_505_ and P_394_U_505_, are illustrated. LCK can phosphorylate itself in *trans* when the catalytic domain of one molecule binds to a tyrosine phosphorylation site on another molecule. Phosphorylated tyrosine residues are red and have a filled red circle labeled with “P”, unphosphorylated sites are green and have an empty red circle. Each LCK species (U_394_U_505_, P_394_U_505_, U_394_P_505_, and P_394_P_505_) is represented by a different color molecule. All of the species can bind to a substrate site (Y394 or Y505) with a single rate of association (*K*_*on*_) and different dissociation rates (*K*_*off,1*_, *K*_*off,2*_, *K*_*off,3*_). The catalytic rates are also different depending on the enzyme and substrate pairs (denoted as *K*_*cat,1*_, *K*_*cat,2*_, *K*_*cat,3*_). (b) Diagram of all possible interactions of the enzyme CSK with LCK. CSK can phosphorylate LCK U_394_U_505_ or P_394_U_505_ on Y505. The pairs can bind with the same association rate (*K*_*on,CSK*_), but CSK-LCK pairs will dissociate (*K*_*off,CSK*-*UU*_, *K*_*off,CSK*-*PU*_) and phosphorylate (*K*_*cat,CSK*-*UU*_, *K*_*cat,CSK*-*PU*_) with different rates.

In the model, each of the four LCK species, referred to by their phosphorylation status as U_394_U_505_, P_394_U_505_, U_394_P_505_, and P_394_P_505_, are able to bind and phosphorylate the four substrate sites, Y394 of U_394_U_505_ and U_394_P_505_ and Y505 of U_394_U_505_ and P_394_U_505_, with different kinetics. To simulate this, the catalytic domain of one LCK species in the model can interact with the Y394 or Y505 residue from another LCK species. This results in several possible dimer conformations between an LCK pair that each has the same association rate (*K*_*on*_), but have different dissociation rates (*K*_*off*_). The phosphorylation reactions can also be catalyzed at different rates depending on both the enzyme and substrate; subsequently, each of the 16 LCK dimer intermediates has a different catalytic rate (*K*_*cat*_). As an illustrative example, the interactions for a representative pair of LCK species, U_394_U_505_ and P_394_U_505_, are shown in Fig. 1a. These two species have 3 different phosphorylation sites, Y394 on U_394_U_505_ and Y505 on U_394_U_505_ and P_394_U_505_, resulting in three different intermediate dimers. After binding with the same rate of association (*K*_*on*_), each of these species can unbind (*K*_*off,1*_, *K*_*off,2*_, *K*_*off,3*_) or catalyze a phosphorylation reaction (*K*_*cat,1*_, *K*_*cat,2*_, *K*_*cat,3*_) with different rates.

Like the LCK homodimers, CSK is able to associate with all of its substrates with the same association rate and has different dissociation and catalytic rates, depending on the substrate (Fig. 1b). It has been widely established in the literature that CSK is only able to phosphorylate LCK at Y505.^41^ Therefore, there are only two substrates available to CSK in the model (U_394_U_505_ and P_394_U_505_), resulting in two intermediate CSK-LCK dimers.

The complete list of interacting pairs in the model and their respective dissociation and catalytic rates is shown in Table 1. In total, the model includes 23 unique species, the concentrations of which are described by 23 ordinary differential equations (ODEs) (Supplemental File 1). The association rate for all LCK pairs (*K*_*on*_) has a median value of 8.9 × 10^−4^ μm^2^/molecules·s and a 90% confidence interval of 6.8 × 10^−4^–1.9 × 10^−3^ μm^2^/molecules·s. The association rate for all CSK-LCK pairs (K_on,CSK_) has a median value of 5.9 × 10^−4^ μm^2^/molecules·s and a 90% confidence interval of 4.4 × 10^−4^–1.2 × 10^−3^μm^2^/molecules·s. The procedure used to estimate these rates is described in detail below.

### Determining the Optimal Parameter Sets

The model was fit to quantitative western blot data of LCK phosphorylation at Y394 and Y505 in a two-dimensional membrane reconstituted system obtained by Hui and Vale.^15^ We used four sets of Y394 and Y505 site-specific phosphorylation data to train the model: 500 molecules LCK/μm^2^, 500 molecules LCK/μm^2^ + 500 molecules CSK/μm^2^, 50 molecules LCK/μm^2^, and 50 molecules LCK/μm^2^ + 500 molecules CSK/μm^2^. A fifth set of site-specific data in which 50% of the LCK in the system is catalytically inactive was used as model validation, to test that the model parameters are able to predict data not used in the fitting process.

As most kinase kinetic studies are performed in solution, we could not directly apply any previous assumptions for the range of parameter values in this two-dimensional system. While there are numerical techniques that enable conversion of three-dimensional kinetic parameters to a two-dimensional system, there is no kinetic binding data of LCK–LCK dimers in either a two-dimensional or three-dimensional system to start from, apart from the autophosphorylation described in the paper by Hui and Vale. Additionally, Hui and Vale compared the autophosphorylation of LCK in solution to their two-dimensional membrane system and found that the phosphorylation kinetics for Y394 and Y505 do not change proportionately when transitioning between the two systems. In the membrane system, Y394 is phosphorylated much faster than Y505, while in solution it appears that Y505 is phosphorylated faster. In the solution data there is an initial rapid jump in Y505 phosphorylation above that of Y394, followed by a plateau and then a second phase in which both sites are rapidly phosphorylated, while in the membrane data the phosphorylation of Y505 is much steadier. These different dynamics imply that it would not be straightforward to inter-convert two-dimensional and three-dimensional kinetics. Therefore, we used an unsupervised fitting procedure in which the parameters are allowed to vary within very wide bounds (10^−20^ to 10^10^ μm^2^/molecules·s for *K*_*on*_, and 10^−20^ to 10^10^ 1/s for *K*_*off*_ and *K*_*cat*_).

**Figure 2:**
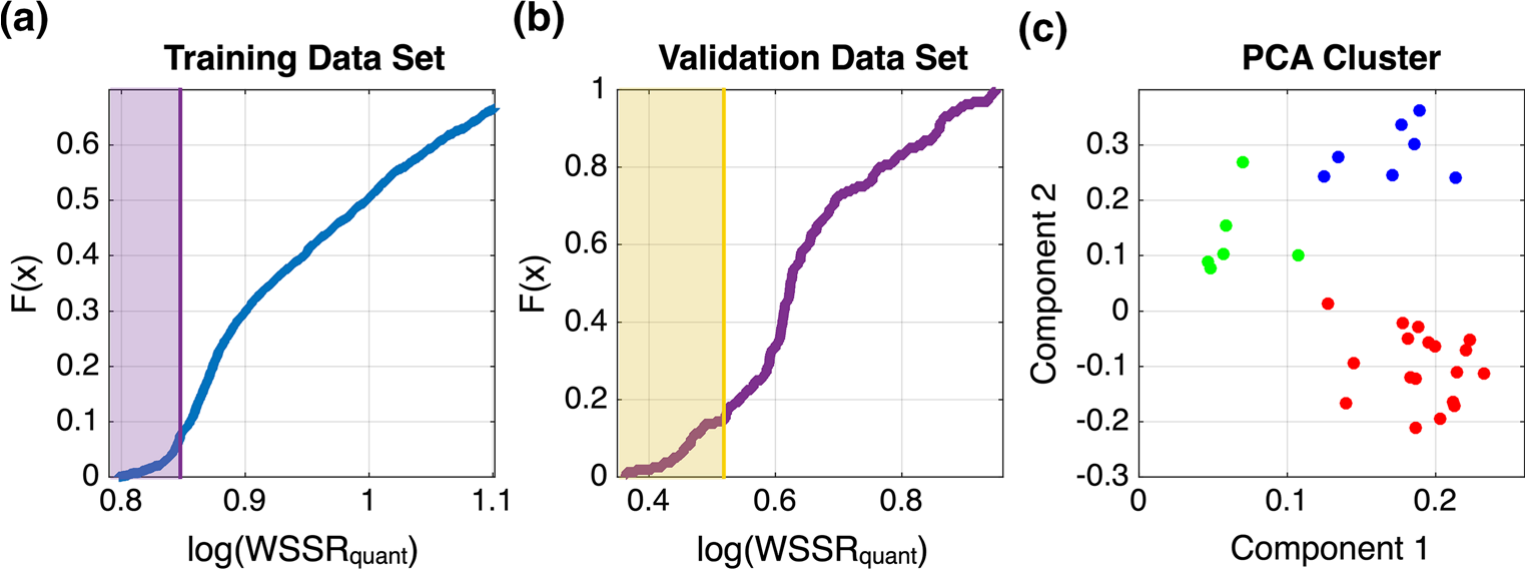
Method for choosing the optimal parameter sets. (a) The cumulative density function of the weighted sum of the squared residuals (WSSR) for training data sets that were used to fit the model. The tail of low WSSR parameter sets was selected (purple region). (b) The parameter sets from the purple region in panel (a) were sorted into a cumulative density function based on the WSSR for the validation data set. Parameter sets with low WSSR were selected (yellow region) and further filtered based on their ability to reach steady state by the end of the 90 min simulation time. (c) The resulting 33 parameter sets were sorted into clusters and compared for trends in their parameter values as well as their ability to fit the training data. The red cluster showed the best fit to the predictive data set, as well as strong statistical differences between many of the parameter values, indicating a clear mechanism of LCK autoregulation.

We used a two-step fitting procedure to search the large parameter space and find parameter sets that can both qualitatively and quantitatively describe the training data. In the first step, we used particle swarm optimization (PSO) to minimize a hybrid weighted sum of the squared residuals (WSSR) objective function.^20^ This hybrid WSSR was used to optimize both the quantitative fit to the data points as well as a qualitative readout of the difference between the curves of phospho-Y394 and phospho-Y505 in each experimental setting (see methods for more detail). We used PSO to obtain 1000 parameter sets that could describe the differences in the rates of phosphorylation of the two sites for the different experimental conditions. PSO is a global optimization technique that enables efficient exploration of the parameter space.^17^ The hybrid WSSR values ranged from 3.3 × 10^1^ to 3.8 × 10^9^, with a median value of 4.1 × 10^2^. However, in the second step, all of the parameter sets obtained using PSO, regardless of their hybrid WSSR value, were tailored to better quantitatively fit the data using the MATLAB lsqnonlin function (MathWorks Inc., Natick, MA). Specifically, the parameter sets from PSO were used as starting points to minimize an objective function that calculates the WSSR between the experimental data and the model predictions. Each of the 1000 PSO parameter sets was tailored twice, resulting in 2000 parameter sets. The frequency distributions of the quantitative WSSR values are shown in Fig. 2a, ranging from 6.3 × 10^0^ to 1.1 × 10^3^.

We used three criteria to determine the optimal parameter sets used for model simulations. Due to the large bounds, the majority of the 2000 parameter sets represented local minima that did not properly capture the data. Therefore, we first considered the parameter sets that were close to a global minimum, with respect to the training data, using the cumulative density function (CDF) of the WSSR. Secondly, we wanted to ensure that the parameter sets chosen were able to predict data not used in the fitting, termed validation data. Therefore, we used the CDF of the WSSR with respect to the validation data to find parameter sets that were able to predict the validation data well. Thirdly, we selected parameter sets that matched a molecularly detailed aspect of the Hui *et al.* data: in the high LCK experimental conditions, nearly 100% of the LCK is doubly phosphorylated by 90 min. To do this, we removed the parameter sets in which less than 90% of the LCK, in the high LCK condition, was doubly phosphorylated by 90 min.

Figures 2a and 2b shows CDF plots for the distributions of the quantitative WSSR values, which are used to find the parameter sets with good fits and predictions, respectively. The general trend is a sigmoidal function, with a tail at the beginning containing the parameter sets that all have low WSSR values. We started with the CDF plot for the training data set, and chose the end of the first step in the function as the cutoff for good fitting parameter sets (Fig. 2a, purple region, WSSR < 7.1). Then, taking only those parameter sets in the purple region, we calculated the WSSR values for the predicted data, which had a WSSR ranging from 2.3 to 8.9. Figure 2b shows the CDF function of these values. A similar cutoff point was chosen for parameter sets that had a good fit to the validation data (WSSR < 3.3), indicated by the yellow region.

The model fitting and parameter selection procedures described above resulted in 33 optimal parameter sets. However, these parameter sets showed high variability, and the median parameter values were not able to reproduce the data. Therefore, we clustered these parameter sets into three groups using the MATLAB kmeans function (Fig. 2c). The three clusters’ predictions of the validation data are shown in Supplemental Fig. 1, with median quantitative WSSR values of 6.8, 7.0, and 6.9 for the green, blue, and red clusters, respectively. These three groups provided different hypotheses for the kinetics of LCK phosphorylation. Although the green cluster had the lowest WSSR, it contained highly variable parameter sets without a significant difference between any of the catalytic rates (data not shown). Without statistical significance, no clear parameter values or mechanistic trends can be observed. The blue and red clusters did show significant differences between the parameters for different LCK species, indicating that specific LCK species interact with different kinetics (Supplemental Fig. 2). The primary difference between the blue and red clusters was the kinetics for the interactions of enzyme P_394_U_505_ with Y505 on U_394_U_505_ and enzyme P_394_P_505_ with Y505 on P_394_U_505_. These differences effectively switched the contribution of these two reactions to the phosphorylation of Y505 in the training data simulations. Significantly, the red cluster had a lower WSSR value, indicating that it was able to match the training data and predict the validation data better than the blue cluster; therefore, the 20 parameter sets within the red cluster were determined to be optimal and were used in subsequent simulations.

### Model Fitting

**Figure 3:**
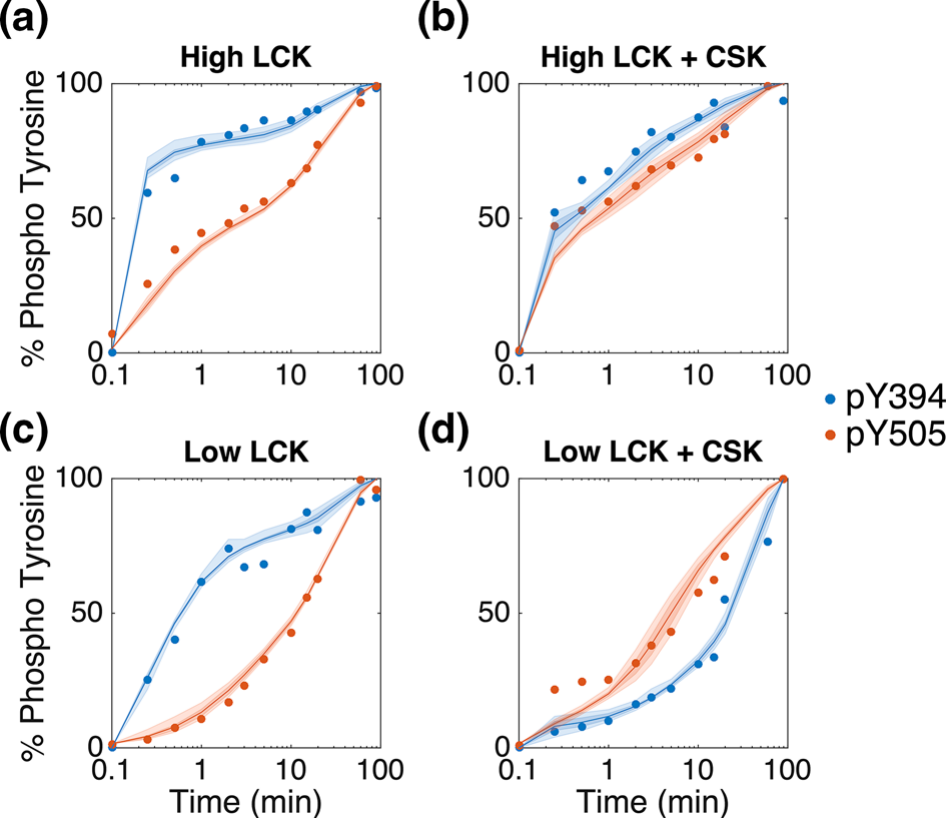
Model fit to experimental data. The model is able to fit experimental data from Hui and Vale.^15^ To mimic the experimental conditions, the model included initial conditions of (a) 500 molecules of LCK/μm^2^, (b) 500 molecules of LCK/μm^2^ + 500 molecules of CSK/μm^2^, (c) 50 molecules of LCK/μm^2^, or (d) 50 molecules of LCK/μm^2^ + 500 molecules of CSK/μm^2^. Each graph shows the experimental data (dots) and median model fit (dark lines) with the 50% and 90% confidence intervals (dark and light shaded regions, respectively). The data shows the total amount of phospho-Y394 (blue) and phospho-Y505 (red) over time based on quantitative western blots. The experimental data is normalized by the western blot band intensity at 90 min, and the simulations are normalized by the concentration of LCK at the end of the 90 min simulation.

Using the 20 optimal parameter sets, the model is able to accurately match the experimental data from Hui and Vale (Fig. 3). In Figs. 3a–3c, LCK Y394 is phosphorylated faster than Y505, and there is more phospho-Y394 in the system than phospho-Y505 at each time point. Conversely, in Fig. 3d, these rates are reversed, and there is more Y505 in the system. The model is able to capture this switch in the rates of Y505 and Y394 phosphorylation. In the high LCK data without or with CSK (Figs. 3a, 3b, respectively), there is a very sharp increase in Y394 phosphorylation within the first 15 s, followed by a much slower increase in phosphorylation. The model is able to capture this biphasic nature as well. Additionally, the model can refine the sharp response in the low LCK + CSK condition, compared to the high LCK conditions (Fig. 3d, blue line).

**Figure 4:**
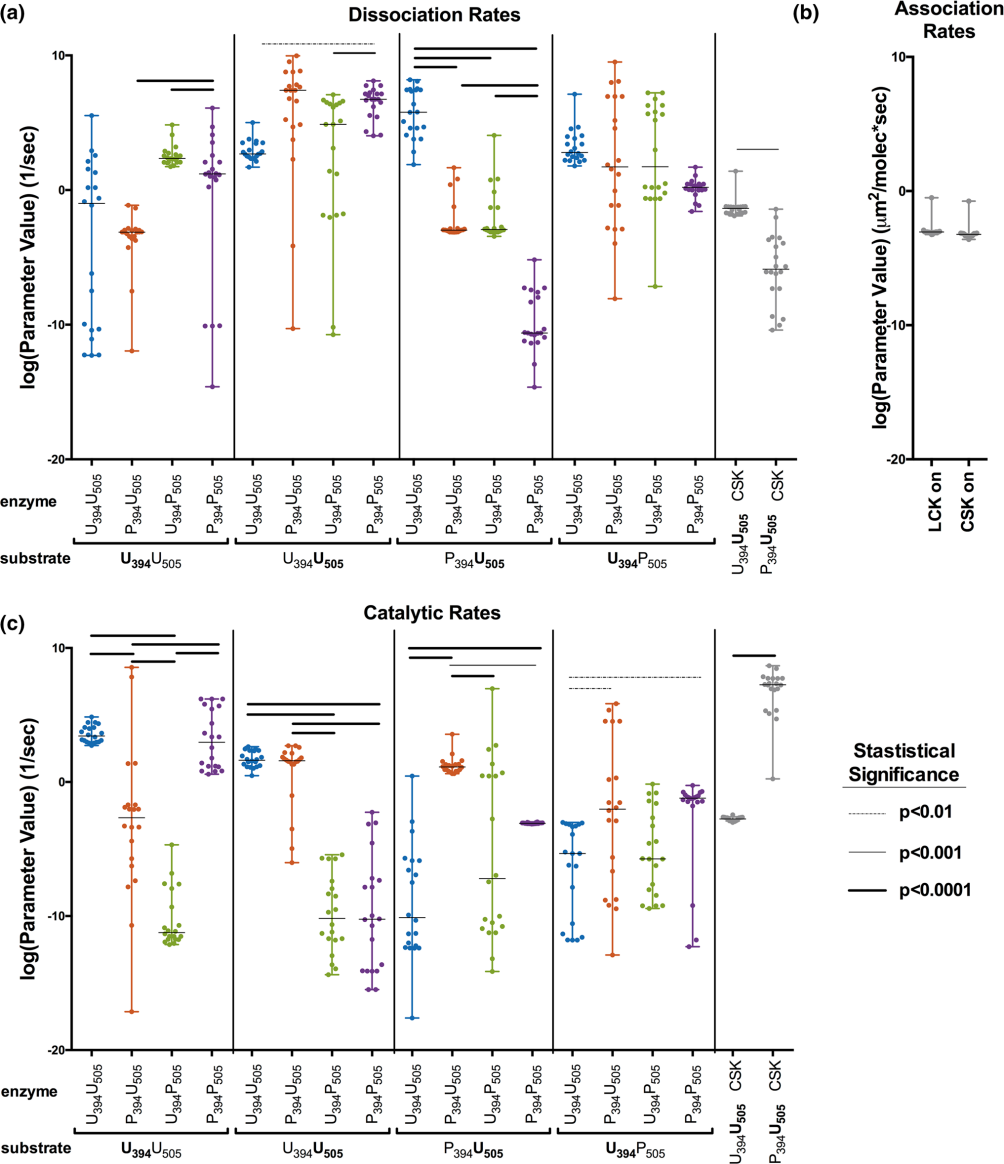
Optimal parameter set values. The distributions for the estimated parameter values are shown for the (a) dissociation rates, (b) association rates, and (c) catalytic rates. The values of the 20 best parameter sets, along with the median and range, are shown. Statistically significant differences between different LCK enzymes acting on the same substrate are denoted by bars above the points, with different thickness representing different levels of statistical significance as calculated by a one-way ANOVA.

There are clear statistical differences between many of the estimated kinetic rates, despite high variability in their fitted values (Fig. 4). The majority of the estimated parameter values vary over a wide range, sometimes over 10 orders of magnitude or more. However, all of the sets are able to reproduce the data used in parameter fitting. Comparing the rates of different enzymes catalyzing the phosphorylation of a single substrate is of particular interest (Figs. 4a and 4c), as these comparisons enable a better understanding of the catalytic activity of individual LCK species. In general, the catalytic rates between enzyme species on a single substrate vary more than the dissociation rates for that substrate. Most of the dissociation rates are relatively high compared to the rate of association, with the exception of the dissociation rate of P_394_P_505_ enzyme with P_394_U_505_ substrate, which is consistently lower than the rate of association. This indicates that the P_394_P_505_-P_394_U_505_ dimer can remain bound for a longer period than most others. The catalytic rate of U_394_P_505_ is always statistically lower than the catalytic rate of all other species. Interestingly, both the LCK and CSK association rates, which are shared between the dimer pairs, are highly conserved, with only one parameter set falling outside of a tight range of less than two-fold (Fig. 4b).

### Model Validation

**Figure 5:**
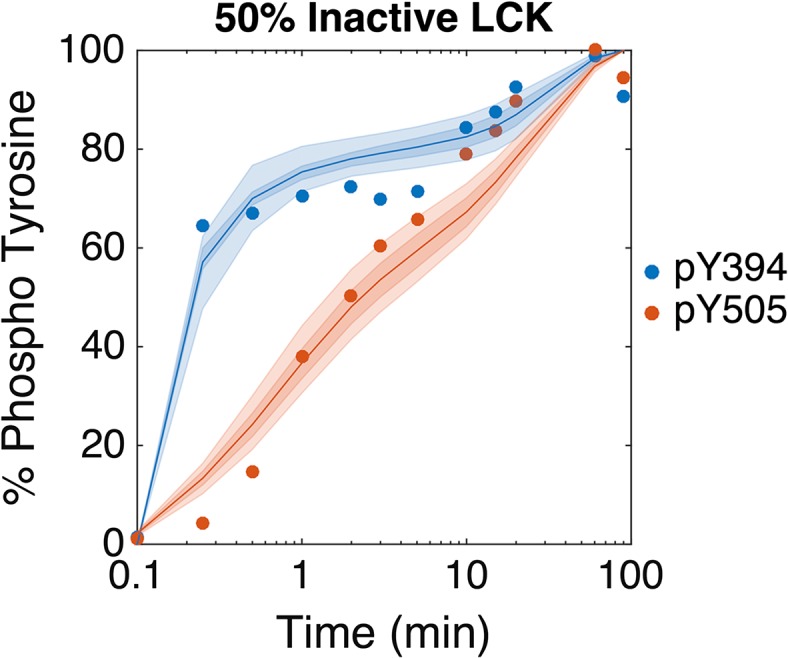
Model validation. The model is able to reproduce data not used in the parameter fitting. This data, taken from Hui and Vale,^15^ uses a reconstituted in vitro membrane system of LCK phosphorylation with a high LCK concentration in which 50% of the LCK (250 molecules LCK/μm^2^) is catalytically inactive due to a point mutation at the ATP binding site and 50% is normally active. The model fit (lines) compared to the data (dots) are shown with 50% and 90% confidence intervals (dark and light shaded areas, respectively), for phospho-Y394 (blue) and phospho-Y505 (red). The experimental data is normalized by the western blot band intensity at 90 min, and the simulations are normalized by the concentration of LCK at the end of the 90 min simulation.

In addition to fitting the training data and estimating the optimal parameter values, the model with the optimal parameter sets is able to match validation data not used in the fitting. Hui and Vale quantified the amount of phospho-Y394 and -Y505 when 50% of the LCK in the system (250 molecules LCK/μm^2^) was made catalytically inactive by a point mutation in the ATP binding site. To simulate this condition, we included a separate LCK species that can be phosphorylated at Y394 and Y505, but cannot act as an enzyme to catalyze the phosphorylation of other molecules. This model assumes that the catalytically inactive LCK interacts with the same kinetic parameters as active LCK. Figure 5 shows that the optimal parameter sets from the model fitting also provide a good match to the experimental validation data not used in the fitting, capturing the sharp early increase in phospho-Y394, the variable slope of phospho-Y505 levels, and the finding that the rate of Y394 is higher than that of Y505. This lends confidence to the predictive ability of the optimized model.

### Sensitivity Analysis

Despite the large variation in many of the parameters, the model is robust and can withstand a high level of biological variability. This is evident in the tight range of model fits and predictions shown in Figs. 3 and 5, respectively (i.e., the small band illustrating the 90% confidence intervals). Additionally, using the median parameter values from all 20 optimal parameter sets, the model is still able to recreate the data within the 50% confidence interval. This indicates significant model robustness, even considering a high level of biological variability.

To further quantify how sensitive the model is to the parameters, we performed a parameter sensitivity analysis using the extended Fourier amplitude sensitivity test (eFAST).^27^ The eFAST analysis is a global variance based method in which all of the parameters are varied together at different frequencies. The Fourier transform of the output can then be analyzed to determine which frequencies, and thus which parameters, have the most influence on the model outputs. This method calculates the first order eFAST indices (*Si*), a measurement of the local sensitivity of each parameter, as well as the total eFAST indices (*STi*), which takes into account the effects of higher order interactions between parameters.

**Figure 6:**
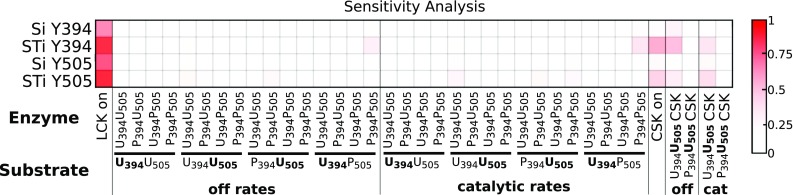
Sensitivity indices of model parameters. The eFAST analysis was used to calculate the first order (*Si*) and total (*STi*) parameter sensitivity indices for two model outputs: total phospho-Y394 (Y394) and total phospho-Y505 (Y505). Red indicates the parameters to which Y394 and Y505 are very sensitive, and white represents parameters that do not significantly influence Y394 and Y505. The dissociation and catalytic rate parameters are labeled by the enzyme-substrate pair involved in the reaction.

Results from the global sensitivity analysis further quantify the robustness of the model. We performed the eFAST analysis to determine the sensitivity of the total phospho-Y394 and total phospho-Y505 in the system, at specific time points, with respect to all 38 kinetic parameters. There were no significant differences between the eFAST results for the high and low concentrations of LCK. Additionally, the qualitative results for the LCK specific parameters did not change with the addition of CSK; therefore, we only show the indices for the condition of high LCK + CSK (Fig. 6). The first order indices were slightly lower than the total indices, but qualitatively the same. The values of the sensitivity indices show that the levels of phosphorylated Y394 and Y505 are most sensitive to the association rates of the LCK species with each other and with CSK, the dissociation rate of CSK and U_394_U_505_, and the catalytic activity of CSK for U_394_U_505_. These are the same parameters for which the estimated values obtained from the model fitting have a narrow distribution. However, the levels of phospho-Y394 and -Y505 are insensitive to most of the parameters, justifying the large deviation in the distributions of the parameters’ estimated values (Fig. 4).

### Predicted Mechanism of LCK Activation

**Figure 7:**
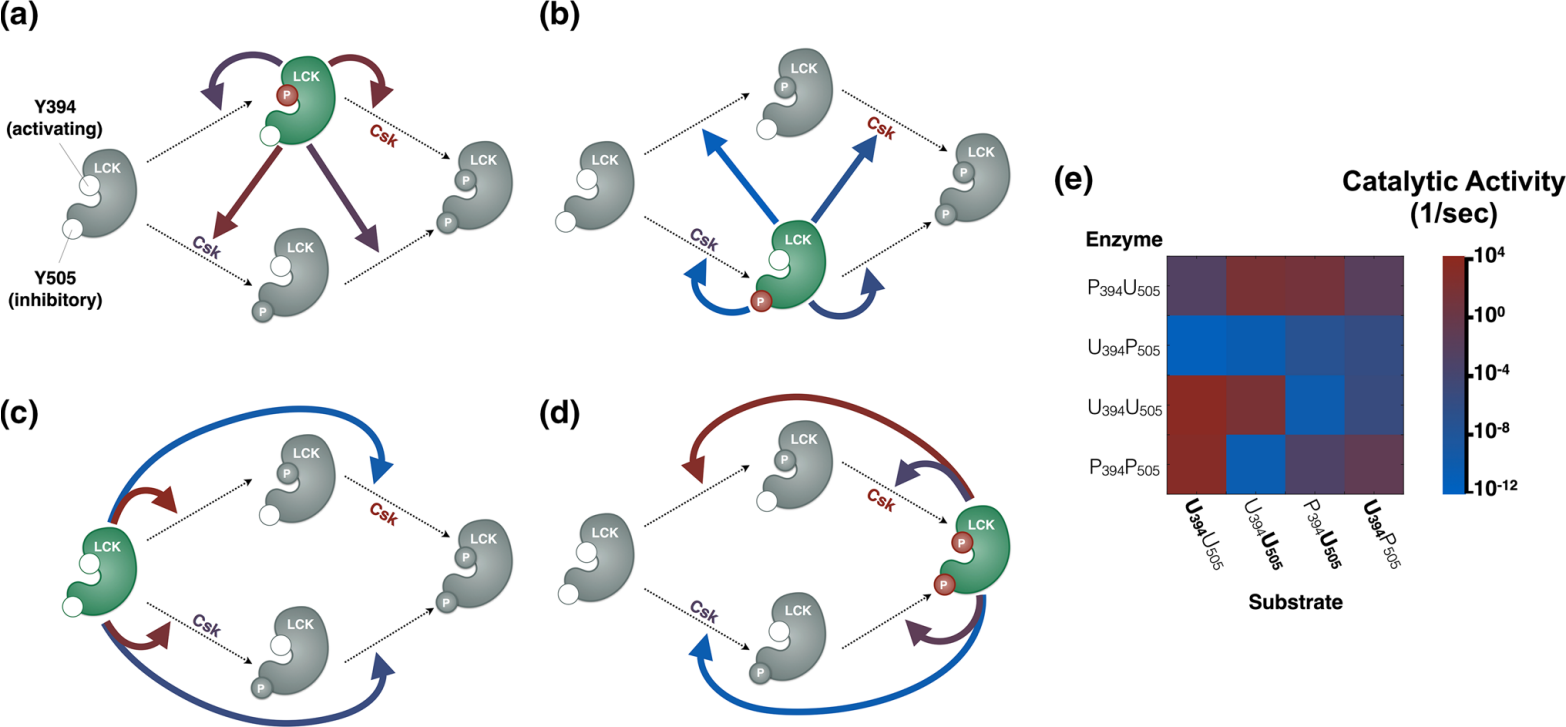
Schematic of predicted LCK kinase activity. The schematics show the catalytic rates for LCK enzymes (a) P_394_U_505_, (b) U_394_P_505_, (c) U_394_U_505_, and (d) P_394_P_505_ catalyzing each of the four possible LCK phosphorylation reactions. In each panel, dotted arrows represent a phosphorylation reaction that can be catalyzed (clockwise from top left, U_394_U_505_→P_394_U_505_, P_394_U_505_→P_394_P_505_, U_394_P_505_→P_394_P_505_, U_394_U_505_→U_394_P_505_). The enzyme catalyzing the reactions in each panel is shown in green with phosphorylated sites shown in red circles. The color of the solid arrows denotes the median value of the catalytic rate for the indicated reaction for the 20 best parameter sets. The reactions catalyzed by CSK are also shown in each panel, with the color of CSK denoting the median value of the CSK catalytic rate. (e) Pairwise heatmap of the LCK enzyme catalytic reactions.

The molecular detail of our model allows us to make predictions regarding the mechanisms of LCK autophosphorylation and phosphorylation by CSK. Specifically, we can compare the median value of the estimated catalytic rates for each of the LCK enzyme species, P_394_U_505_, U_394_P_505_, U_394_U_505_, and P_394_P_505_, as shown in Figs. 7a, 7b, 7c, and 7d, respectively, with a summary of the pairwise interactions shown in Fig. 7e. The parameter estimation reveals that P_394_U_505_ has the highest overall catalytic activity (i.e., this enzyme species only has red or purple arrows pointing to the phosphorylation reactions, Fig. 7a). Conversely, U_394_P_505_ has the lowest activity (Fig. 7b). These results are in agreement with what has been shown in the literature for the catalytic rates of these enzymes on other substrates, such as the CD3z chain ITAMs.^9^,^15^ The catalytic activity of the U_394_U_505_ and P_394_P_505_ species varies depending on the substrate. U_394_U_505_ preferentially phosphorylates itself and catalyzes phosphorylation at Y394 and Y505 with approximately the same rate. In comparison, P_394_P_505_ shows a strong preference for site Y394 of U_394_U_505_. CSK is estimated to have higher catalytic activity against P_394_U_505_ than U_394_U_505_, predicting that CSK will phosphorylate P_394_U_505_ more readily than U_394_U_505_, which has been validated experimentally.^5^,^41^

The estimated catalytic activities identify specific molecular interactions that produce a negative feedback loop in LCK activation. The P_394_U_505_ species, the most catalytically active form of the LCK, preferentially phosphorylates Y505, compared to Y394, with a difference of over three orders of magnitude (Fig. 7a, red arrows). This is significant because Y505 is thought to be the inhibitory site, generally, and its phosphorylation reduces the catalytic activity of LCK, thus providing a possible form of negative feedback. Doubly phosphorylated LCK, P_394_P_505_, on the other hand, preferentially phosphorylates U_394_U_505_ to P_394_U_505_, increasing the overall catalytic activity of the pool of LCK. It has been shown that other intermolecular feedback mechanisms do play an important role in controlling and tailoring the T cell response^42^; however, these specific autoregulatory feedback mechanisms have not been identified before. Thus the model predicts, for the first time, that competing intramolecular feedback loops could help stabilize and control the overall activity of LCK. It will be important to see if these effects are still significant when more complex interactions between phosphatases and other substrates are taken into account.

**Figure 8:**
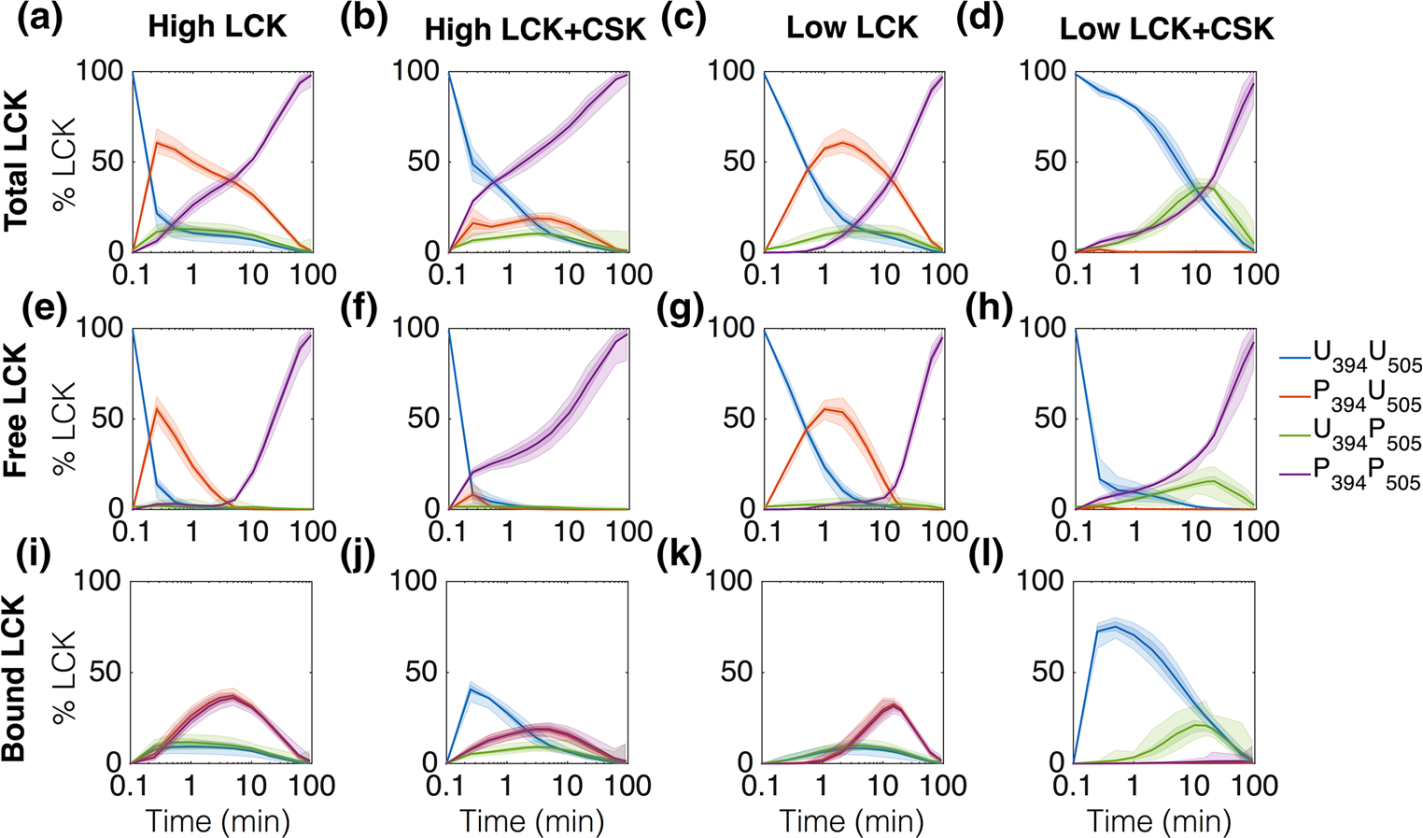
Model predictions of intermediate LCK species. The graphs represent the model simulations for total (a-d), free (e–h), and bound (i-l) LCK species over time. From left to right, the columns represent data from conditions of 500 molecules of LCK/μm^2^, 500 molecules of LCK/μm^2^ + 500 molecules of CSK/μm^2^, 50 molecules of LCK/μm^2^, and 50 molecules of LCK/μm^2^ + 500 molecules of CSK/μm^2^. The results are shown as a percentage of the total LCK in the system, with the 50% and 90% confidence intervals (dark and light shaded regions, respectively).

The model also predicts the relative amounts of each LCK species in the system over time. In general, LCK starts in a completely unphosphorylated form and transitions through a singly phosphorylated intermediate to end with all of the LCK doubly phosphorylated (Fig. 8). The results are shown for both high and low LCK concentrations with and without CSK.

The predicted levels of the LCK species are directly influenced by the binding kinetics. The model predicts that a significant amount of P_394_U_505_ and P_394_P_505_ can remain bound together when they are both present in the system (Figs. 8i and 8k). This is due to the low dissociation rate for enzyme P_394_P_505_ bound to substrate P_394_U_505_ (Fig. 4a). U_394_P_505_ and U_394_U_505_ are also able to bind together, but this association is not as strong as that of P_394_P_505_ and P_394_U_505_ and seems to be primarily a result of the low catalytic activity for all binding conformations of these pairs. A similar, but even smaller, binding interaction is present between U_394_P_505_ and P_394_U_505_. When CSK is added to the system, it serves as a sink for LCK, binding to it and keeping it in the system for longer. Significantly, much more U_394_U_505_ is bound to CSK than P_394_U_505_, as shown by the sharp increase in bound U_394_U_505_ when CSK is present (comparing Figs. 8l to 8k, blue lines), compared to the more modest increase in P_394_U_505_ (comparing Figs. 8l to 8k, green lines).

The model allows us to explore the detailed mechanisms that govern previously unexplained features of the data. The presence of CSK significantly influences the levels of the LCK species. Adding CSK to the system greatly reduces the total amount of P_394_U_505_, leaving almost none of this highly active form free to interact with other species (comparing Figs. 8h to 8g, red lines). In the low LCK conditions, CSK also increases the amount of U_394_P_505_, greatly reducing the overall catalytic activity of the total pool of LCK in this experimental condition (comparing Figs. 8d to 8c, green lines). In comparison, in the high LCK + CSK experimental simulation, the total amount of U_394_P_505_ does not significantly change compared to the high LCK condition (comparing Figs. 8b to 8a, green lines), while the amount of P_394_U_505_ is still greatly reduced (Figs. 8b to 8a, red lines). The model predicts that this difference in the change of intermediate U_394_P_505_ is responsible for the shift in the curves of phospho-Y394 and phospho-Y505 in Fig. 3d of the model training data sets.

## Discussion and Conclusion

We have constructed a model of LCK activation *via* autophosphorylation and phosphorylation by the kinase CSK based on data from an *in vitro* two-dimensional membrane system.^15^ LCK is an important regulator of T cell activation, and quantifying the kinetics that govern its activity will allow us to better understand and engineer T cells for therapeutic purposes. The kinetics of LCK phosphorylation in this *in vitro* membrane system are very different from those that occur in more commonly used solution systems. It is believed that this two-dimensional system more accurately reflects what occurs *in vivo*, as much of the LCK in T cells is bound to the inside of the membrane.^46^ Most prior biological computational signaling models have relied on enzyme solution kinetic parameters for initial estimates. However, we believe that by focusing on two-dimensional kinetics that are more representative of what occurs *in vivo*, we can create models that are more predictive.

Our model is able to fit the data of membrane bound LCK phosphorylation well, both quantitatively and qualitatively. Additionally, we are able to identify the specific kinetic parameters that most significantly control LCK phosphorylation. One limitation of the model is its inability to match early time point experimental measurements of the phospho-Y505 curve in the low LCK + CSK experimental condition (Fig. 3d). However, there are no error bars for the experimental data, and it is possible that these data may have some experimental error. The recombinant LCK protein used in this system is autophosphorylated as it is expressed, so it must first be dephosphorylated before the start of the experiment. Hui *et al.* used mass spectrometry to measure the efficiency of this dephosphorylation and found that a small amount of Y505 is still phosphorylated at the start of the experiment (~1.5%); however, many graphs show that the initial phosphorylation of Y505 is much higher than that, up to ~20%. This large variability in the starting concentration of phospho-Y505 could lead to an overestimation of the initial rate of Y505 phosphorylation in the low LCK + CSK condition. Additionally, the data are derived from quantitative western blotting, and there may be error in the band intensity readings, particularly for early time points where the band intensity is very close to background. For these reasons, it is possible that the initial increase in phospho-Y505 in the low LCK + CSK condition does not truly reflect LCK kinetics.

We applied an unbiased approach to fit the parameters, generating a set of optimal parameter values that are able to reproduce the data. Despite high variability in the fitted parameters, there are statistically significant differences between the estimated dissociation rates of the LCK dimers and catalytic activities of the LCK enzymes. The statistical analysis along with the global sensitivity analysis indicate that the proposed mechanism of LCK activation implemented in the model is robust and predictive. The model predictions reveal that the levels of individual LCK species can remain within a tight range, even with high variability in the parameter rates. This model robustness is biologically relevant, since it has been shown that the local microenvironment around a pool of LCK in the cell changes dramatically depending on the state of the cell and the proximity of other molecules.^12^,^16^,^19^,^35^

The model brings many new insights into the autoregulatory mechanisms of LCK with respect to the binding of LCK dimers. For example, the model predicts that P_394_U_505_ and P_394_P_505_ are able to form a relatively strong dimer compared to P_394_U_505_-U_394_P_505_ or U_394_U_505_-U_394_P_505_. Additionally, there are pairs that do not significantly dimerize at all. A crystal structure of the LCK SH2 and SH3 domains show that these domains can homodimerize, and that this binding may be stabilized by the addition of the phosphorylated Y505 tail.^10^ However, it does not provide any information about how interactions from other domains, particularly the domain containing Y394, control the extent of this dimerization. The model parameters specify which dimers are able to bind more strongly, and from that, we can infer the role that these other domains play in LCK binding. Since the P_394_U_505_-P_394_P_505_ dimer is stronger than P_394_U_505_-U_394_P_505_, we can hypothesize that the phospho-Y505 tail of P_394_P_505_ may be more amenable to stabilizing the P_394_U_505_-P_394_P_505_ dimer interaction than that of U_394_P_505_. This may be because the phospho-Y394 in P_394_P_505_ keeps the molecule in a partially open conformation while the tail of U_394_P_505_ is held in a closed conformation through *cis* binding.^9^ Also, since U_394_U_505_ and P_394_U_505_ do not dimerize with themselves or each other, we can conclude that the stabilization from the phospho-Y505 tail is important in the LCK intermolecular interactions.

The model also predicts that CSK plays a very strong role in controlling the distribution of LCK species in the model, which could be important for controlling LCK activity *in vivo*. Figure 8 shows that CSK is able to bind to LCK and increase the amount of U_394_U_505_ and U_394_P_505_ in the system while reducing the amount of P_394_U_505_ and P_394_P_505_. It is known that clusters of T cell signaling molecules reside close to each other on lipid rafts inside the T cell membrane.^12^,^19^ The composition of these clusters changes as the T cell becomes activated and the immunological synapse begins to form.^16^ Keeping LCK clustered with CSK before synapse formation could act as a control mechanism to reduce aberrant LCK signaling in unstimulated cells. Once the synapse forms and CSK is sequestered outside of the synapse region, enough LCK can accumulate to lead to high levels of active P_394_U_505_. More studies need to be done to better understand how the possible LCK autoregulatory feedback mechanisms and CSK function *in vivo* when there are more substrates and phosphatases in the system.

The model also predicts new binding relationships between CSK and LCK that have not been identified experimentally. The model indicates that CSK is able to bind more strongly to U_394_U_505_ than to P_394_U_505_ (Fig. 8l). Conversely, studies of LCK binding to CSK in solution have shown that CSK is able to bind to P_394_U_505_, but not U_394_U_505_.^5^ Combined, these results suggests that CSK binding to U_394_U_505_ could be an effect of the two-dimensional membrane system, indicating a significant difference between the mechanisms that occur in solution and those that are able to take place in a more physiologically relevant membrane bound arrangement.

Comparing the model simulations to data from LCK phosphorylation in solution continues to shed light on the differences between studying molecular kinetics in solution and in the native two-dimensional distribution. Hui *et al.* compared their experimental membrane system to a traditional solution system. The authors found that the rates at which the two LCK sites were phosphorylated were significantly different, and that the measured kinetics for levels of phospho-Y394 and -Y505 did not change proportionately. In the membrane system, for high LCK, Y394 is rapidly phosphorylated while Y505 slowly increases in a more steady manner (Fig. 3a). In solution, however, Y394 and Y505 phosphorylation both remain at their starting levels for about 10 min and then both increase very rapidly.

The model indicates that distinct mechanistic interactions can potentially contribute to differences in the LCK phosphorylation kinetics that occur in two-dimensions compared to solution. Our model and estimated parameter sets were obtained by fitting LCK phosphorylation data from the *in vitro* reconstituted membrane system developed by Hui and Vale.^15^ We also attempted to fit data for LCK phosphorylation measure in solution. Since a key distinction between the two-dimensional and solution-based systems is that the species’ amounts are given in units of density rather than concentration, we attempted to fit the in solution data by only adjusting the association rates. The association rates are the only parameters that depend on the amount of a species (i.e., *K*_*on*_ has units of μm^2^/molecules·s), whereas the dissociation and catalytic rates do not depend on concentration. We followed the same parameter fitting procedure described above using each of the membrane bound optimal parameter sets described in the paper as starting values to minimize the quantitative WSSR equation with the MATLAB lsqnonlin function; however, we were unable to fit the solution data. We then expanded our fitting of the solution data to include the association and dissociation rates, or the association, dissociation, and catalytic rates. The data still could not be fit with the mechanism used in the model. Although more experiments need to be done to properly compare the differences between LCK in solution and on the two-dimensional membrane surface, we believe this could point to a difference not only in the parameter values but also in the mechanism of LCK phosphorylation between the two settings.

Excitingly, the fitted model generates testable hypotheses, and the experimental *in vitro* two-dimensional membrane system can be used to explore some of these model predictions. The estimated parameter values and model predictions support the presence of both negative and positive autoregulated feedback on the catalytic activity of LCK, which have not been described previously. The negative feedback comes from catalytically active P_394_U_505_ preferentially phosphorylating other LCK molecules at the Y505 inhibitory site. The positive feedback comes from the moderately active P_394_P_505_ species preferentially pushing doubly unphosphorylated LCK to the active P_394_U_505_ form (Figs. 7a and 7d). These new feedback mechanisms, hypothesized by the model predictions, can be tested with targeted experiments that focus specifically on the catalytic activities of individual phospho-LCK species. We can do this by mixing LCK that is either doubly phosphorylated or specifically phosphorylated only at Y394 with other, catalytically inactive, LCK species. It is also possible to test model hypotheses about the significance of bound dimers, like CSK and U_394_U_505_, by inserting domain deficient mutants, such as LCK lacking the SH2 or SH3 domains, into the two-dimensional membrane system. Thus, a systems biology approach of using an optimized and validated computational model in combination with quantitative experimental approaches can provide new and relevant biological insight into LCK activation.

It is possible to improve and strengthen the model by adding new proteins into this same *in vitro* membrane reconstituted system and performing model parameter estimation, as we have done here. This will allow us to better understand how individual proteins combine to produce the functions of the system as a whole. For example to better understand the mechanisms of LCK activation, we can incorporate dephosphorylating events, through proteins like CD45 and PTPN22, into the model to see how that action impacts the overall levels of individual LCK species.^28^ Having more data to fit the model will also help to more specifically identify the LCK kinetic parameters, many of which are still highly variable in the current model. We can also study more specific mechanisms of LCK by adding its substrates, CD3ζ, ZAP-70, and SHP-1 into the system. The model also serves as a starting point for studying the order and kinetics of LCK-mediated phosphorylation of the six CD3ζ ITAM tyrosine phosphorylation sites.^8^,^21^,^43^ We believe that the model provides a quantitative framework for studying many different protein interactions relevant to T cell signaling, particularly those involving LCK.

In summary, the model is a predictive tool that can be used to examine the dynamics of LCK autoregulation. As we continue to expand the model, we can use it to make new predictions about the larger systems that govern T cell activation and explore key biological hypotheses, like those described above. Many of the mechanistic questions described in this paper have proven difficult to investigate experimentally; however, using the computational framework described here, we will be able to explore these issues on a more quantitative level, providing insights and new testable hypotheses.

## Electronic supplementary material

Below is the link to the electronic supplementary material.

Supplemental File 1: Ordinary Differential Equations. This file lists the 23 ordinary differential equations from the model of LCK + CSK, as well as a table of the symbols representing each species described by the ODEs. Supplementary material 1 (TIFF 199216 kb)

Supplemental File 2: LCK + CSK Model BioNetGen Source Code. BioNetGet source code to generate the MATLAB executable ODEs for the model of LCK + CSK in a two-dimensional reconstituted membrane system.. Supplementary material 2 (TIFF 84183 kb)

Supplemental File 3: 50% Inactive LCK Model BioNetGen Source Code. BioNetGet source code to generate the MATLAB executable ODEs for the model of 50% inactive LCK + 50% wild type LCK in a two-dimensional reconstituted membrane system. Supplementary material 3 (PDF 44 kb)

Predictions for three parameter set clusters. Results of model validation of the three different parameter clusters, green (a), blue (b), and red (c), from Figure 2c. This validation data, taken from the literature (Hui, 2014), uses a reconstituted in vitro membrane system of LCK phosphorylation in which 50% of the LCK is catalytically inactive due to a point mutation at the ATP binding site and 50% is normally active. The model fits (lines) to the data (dots) are shown with 50% and 90% confidence intervals (dark and light shaded areas, respectively), for phospho-Y394 (green) and phospho-Y505 (purple). The experimental data is normalized by the western blot band intensity at 90 minutes, and the simulations are normalized by the concentration of LCK at the end of the 90 minutes simulation. Supplementary material 4 (BNGL 7 kb)

Comparison of Red Cluster and Blue Cluster Parameters. The comparison of estimated parameter sets for the blue and red cluster of Figure 2c are shown for the (a) binding parameters, (b) catalytic parameters, and (c) CSK parameters. The data show the values of the parameter sets with the median and range. Statistically significant differences between the clusters are denoted by bars above the points, with different numbers of stars representing different levels of statistical significance as calculated by a one-way ANOVA. Supplementary material 5 (BNGL 14 kb)
