# Supplementary material for: Predictive Model of Lymphocyte-Specific Protein Tyrosine Kinase (LCK) Autoregulation
Source: Cell Mol Bioeng. 2016 Apr 26;9:351–67. doi: 10.1007/s12195-016-0438-7 (PMC4978775; doi:10.1007/s12195-016-0438-7)
Supplement: Supplementary file 3 — Supplemental File 3: 50% Inactive LCK Model BioNetGen Source Code. BioNetGet source code to generate the MATLAB executable ODEs for the model of 50% inactive LCK + 50% wild type LCK in a two-dimensional reconstituted membrane system. Supplementary material 3 (PDF 44 kb) [file 12195_2016_438_MOESM3_ESM.pdf]

ds1/dt = -kon*s1*s11 +kcat3*s13 -kon*s1*s12 +kcat1*s4 -kon*s1*s11 +koff4*s14 +2.0*koff1*s4 -konCSK*s3*s1 +koff7*s7 +kcat2*s5 +koff8*s8 -kon*s1*s2 +koff13*s16 -2.0*kon*s1*s1 +2.0*koff2*s5 -2.0*kon*s1*s1 +koffCSKUU*s10 +koff3*s13 -kon*s1*s2 +koff14*s17 -kon*s1*s12 -kon*s1*s2 +koff5*s15 -kon*s1*s11 +koff6*s6 +kcat6*s6 ;
ds2/dt = -kon*s1*s2 +koff8*s8 +kcat2*s5 +kcat12*s9 +koff11*s21 +koff7*s7 +koff10*s20 +koff16*s22 +kcatCSKUU*s10 +2.0*kcat8*s8 +kcat5*s15 +koff6*s6 +2.0*koff12*s9 -kon*s1*s2 -kon*s2*s12 -2.0*kon*s2*s2 -kon*s11*s2 -kon*s1*s2 +kcat14*s17 -kon*s11*s2 +kcat10*s20 +kcat7*s7 ;
ds3/dt = koffCSKUU*s10 -konCSK*s3*s11 +kcatCSKUU*s10 +kcatCSKPU*s23 -konCSK*s3*s1 +koffCSKPU*s23 ;
ds4/dt = -kcat1*s4 +kon*s1*s1 -koff1*s4 ;
ds5/dt = -koff2*s5 -kcat2*s5 +kon*s1*s1 ;
ds6/dt = -koff6*s6 -kcat6*s6 +kon*s1*s2 ;
ds7/dt = kon*s1*s2 -kcat7*s7 -koff7*s7 ;
ds8/dt = -kcat8*s8 -koff8*s8 +kon*s1*s2 ;
ds9/dt = -koff12*s9 -kcat12*s9 +kon*s2*s2 ;
ds10/dt = konCSK*s3*s1 -kcatCSKUU*s10 -koffCSKUU*s10 ;
ds11/dt = -kon*s11*s12 +kcat9*s18 +2.0*kcat4*s14 +kcat1*s4 -kon*s1*s11 -kon*s1*s11 +koff11*s21 +2.0*koff9*s18 +kcat11*s21 -konCSK*s3*s11 +koff10*s20 +koff4*s14 +kcat5*s15 -2.0*kon*s11*s11 +koff5*s15 -kon*s1*s11 -kon*s11*s2 +koffCSKPU*s23 +kcat13*s16 +koff3*s13 +koff15*s19 -kon*s11*s2 +kcat7*s7 ;
ds12/dt = kcat14*s17 +kcat10*s20 +koff15*s19 +kcat13*s16 -kon*s2*s12 +kcat6*s6 -kon*s1*s12 +koff14*s17 +koff16*s22 +kcat11*s21 +2.0*kcat16*s22 +kcat3*s13 -kon*s1*s12 +2.0*kcat15*s19 +kcatCSKPU*s23 +kcat9*s18 +kcat12*s9 -kon*s11*s12 +koff13*s16 ;
ds13/dt = -kcat3*s13 +kon*s1*s11 -koff3*s13 ;
ds14/dt = kon*s1*s11 -kcat4*s14 -koff4*s14 ;
ds15/dt = -koff5*s15 +kon*s1*s11 -kcat5*s15 ;
ds16/dt = -kcat13*s16 +kon*s1*s12 -koff13*s16 ;
ds17/dt = -kcat14*s17 +kon*s1*s12 -koff14*s17 ;<br>ds18/dt = -kcat9*s18 +kon*s11*s11 -koff9*s18 ;
ds19/dt = kon*s11*s12 -koff15*s19 -kcat15*s19 ;

ds20/dt = -koff10*s20 -kcat10*s20 +kon*s11*s2 ;
ds21/dt = -koff11*s21 +kon*s11*s2 -kcat11*s21 ;
ds22/dt = kon*s2*s12 -koff16*s22 -kcat16*s22 ;
ds23/dt = -koffCSKPU*s23 -kcatCSKPU*s23 +konCSK*s3*s11 ;

**Free**	**Symbol**	**Bound**		**Symbol**
		Enzyme	Substrate	
$U_{394}U_{505}$	S_1	$U_{394}U_{505}$	$\mathbf{U_{394}}U_{505}$	S_4
$P_{394}U_{505}$	S_{11}	$U_{394}U_{505}$	$U_{394}\mathbf{U_{505}}$	S_5
$U_{394}P_{505}$	S_2	$U_{394}U_{505}$	$P_{394}\mathbf{U_{505}}$	S_{13}
$P_{394}P_{505}$	S_{12}	$P_{394}U_{505}$	$\mathbf{U_{394}}U_{505}$	S_{14}
CSK	S_3	$P_{394}U_{505}$	$U_{394}\mathbf{U_{505}}$	S_{15}
		$U_{394}U_{505}$	$\mathbf{U_{394}}P_{505}$	S_6
		$U_{394}P_{505}$	$\mathbf{U_{394}}U_{505}$	S_7
		$U_{394}P_{505}$	$U_{394}\mathbf{U_{505}}$	S_8
		$P_{394}U_{505}$	$P_{394}\mathbf{U_{505}}$	S_{18}
		$U_{394}P_{505}$	$P_{394}\mathbf{U_{505}}$	S_{20}
		$P_{394}U_{505}$	$\mathbf{U_{394}}P_{505}$	S_{21}
		$U_{394}P_{505}$	$\mathbf{U_{394}}P_{505}$	S_9
		$P_{394}P_{505}$	$\mathbf{U_{394}}U_{505}$	S_{16}
		$P_{394}P_{505}$	$U_{394}\mathbf{U_{505}}$	S_{17}
		$P_{394}P_{505}$	$P_{394}\mathbf{U_{505}}$	S_{19}
		$P_{394}P_{505}$	$\mathbf{U_{394}}P_{505}$	S_{22}
		CSK	$U_{394}\mathbf{U_{505}}$	S_{10}
		CSK	$P_{394}\mathbf{U_{505}}$	S_{23}
